# Editorial: Alternative Therapeutics Against Antimicrobial-Resistant Pathogens

**DOI:** 10.3389/fmicb.2019.02173

**Published:** 2019-09-19

**Authors:** Rebecca Thombre, Kamlesh Jangid, Ravi Shukla, Noton Kumar Dutta

**Affiliations:** ^1^Department of Biotechnology, Modern College of Arts, Science and Commerce, Pune, India; ^2^School of Physical Sciences, University of Kent, Canterbury, United Kingdom; ^3^National Centre for Microbial Resource, National Centre for Cell Science, Savitribai Phule Pune University Campus, Pune, India; ^4^NanoBiotechnology Research Laboratory, RMIT University, Melbourne, VIC, Australia; ^5^Center for Tuberculosis Research, Department of Medicine, Johns Hopkins University School of Medicine, Baltimore, MD, United States

**Keywords:** antimicrobial resistance (AMR), multidrug-resistance (MDR), nanomaterials, nanoparticles, plant based compounds, antimicrobial agents, alternative therapy, novel compounds

Antimicrobial resistance (AMR) has emerged as one of the greatest global challenge to public health in the twenty-first century. The use of antibiotics is associated with the serendipitous discovery of Penicillin by Sir Alexander Fleming in 1928 (Fleming, 1929). However, Emmerich and Löw had demonstrated the first application of the antibiotic “Pyocyanase” in hospital in 1899. The golden era of antibiotic was ushered between 1950s and 1970s which was marked by rapid developments in discoveries of many classes and types of antibiotics (Emmerich and Löw, 1899). The increased use and abuse of antibiotics caused the emergence of multidrug resistant bacteria that caused hard-to-treat infections (Aminov, 2010). It is imperative to search alternative therapeutics and strategies to combat AMR and diminish the exacerbated use of antibiotics. In this special issue, we present 26 articles that highlight the use of novel peptides, phage-based therapies, nanomedicine, contemporary and alternative medicines, plant (herbal), and bacteria based antimicrobials as potential alternatives to combat multidrug resistant (MDR) bacteria. The articles are categorized in different groups, including (i) Antimicrobial nanoparticles against drug resistant bacteria, (ii) Bacteriophages: A promising approach to fight MDR, (iii) Anti biofilm agents, (iv) Antimicrobial peptides, (v) Efflux pump inhibitors, and (vi) Host /Pathogen directed therapies.

A variety of repurposing FDA-approved drugs (Sharma et al.) that are employed in the management of pathological conditions of non-infectious etiology have been shown to exhibit broad spectrum antimicrobial activity *in vitro* and *in vivo*. Such compounds including marine eukaryotes like seaweeds (Karthick and Mohanraju), phytochemicals (Kim et al.; Lu et al.), antimicrobial peptides and proteins (Kumar et al.), termed “non-antibiotics” (Dutta et al., 2007; Mazumdar et al., 2009, 2010), possess antibacterial properties, acting through mechanisms different from those of existing drugs, by enhancement of combination-therapy effective (Pachon-Ibanez et al.; Shriram et al.), reversal of drug resistance (Guo et al.; Patwardhan et al.) or re-sensitizing activities (Dutta et al., 2014; Shriram et al.), inhibition of biofilm formation (Guo et al.; Karumathil et al.; Kaur et al.; Khalifa et al.; Kim et al.; Lu et al.; Punjabi et al.), as well as by induction and control of efflux pumps (Dutta et al., 2010; Baptista et al.; Karumathil et al.; Lu et al.; Shriram et al.).

This broad group of antimicrobial agents has two sub-groups, each with distinctly different adjunct activities, either pathogen-directed or host directed. The first group is that of the antimicrobial non-antibiotics—drugs that have direct antimicrobial activity and the proposed path for compounds targeting microbial factors. The second group can generally be classified into two categories: those that enhance the antimicrobial activity of the host immune system, and those which dampen the inflammatory response preventing tissue damage (Karumathil et al.; Singh and Subbian). Host-directed therapies are attractive options as they are not prone to the resistance associated with antibiotics (Dutta et al., 2016; Frank et al., 2019). Currently, the following HDT agents are being evaluated in phase 2 clinical trials as adjuncts to rifabutin-modified standard therapy in adults with drug-sensitive, smear-positive pulmonary TB: (1) the mammalian target of rapamycin (mTOR) inhibitor, everolimus (0.5 mg), (2) auranofin (6 mg), (3) vitamin D3, and (4) the phosphodiesterase-4 (PDE4) inhibitor, CC-11050 (ClinicalTrials.gov Identifier: NCT02968927). A randomized clinical trial, Statins as Adjunctive Therapy for TB (StAT-TB), is currently underway to determine if pravastatin adjunctive therapy shortens the median time to sputum-culture negativity and improves lung function outcomes among HIV-infected and uninfected patients with drug-susceptible pulmonary TB (NCT03456102).

In summary, these articles cover a vast expanse of research findings based on emerging trends in combatting antimicrobial resistance using traditional and natural antimicrobials, plant and microbial derivatives and nanomaterials. Currently, AMR is a constantly growing global threat to public health worldwide and has been declared as a thrust area by World Health Organization (WHO). AMR is mediated via various mechanisms such as enzymatic degradation of drugs, alteration of antimicrobial targets, efflux of drugs, alteration of microbial membrane permeability, formation of biofilms, and persister cell states. Most of the AMR resistance genes (ARG) are disseminated via horizontal gene transfer mediated by genetic elements like plasmids, transposons, bacteriophages, and other genetic elements (Thombre et al., 2016). One of the challenges of AMR is annihilating the spread and prevalence of ARGs in the environmental resistome via plasmids. Future strategies and new lines of research need to be undertaken using conjugation inhibitors, plasmid incompatibility systems, and CRISPR/Cas-based approaches to tackle the incredibly profound multidrug resistant bacteria (Buckner et al., 2018).

## Author Contributions

All authors listed have made a substantial, direct and intellectual contribution to the work, and approved it for publication.

### Conflict of Interest

The authors declare that the research was conducted in the absence of any commercial or financial relationships that could be construed as a potential conflict of interest.
